# Docosahexaenoic Acid-Loaded Polylactic Acid Core-Shell Nanofiber Membranes for Regenerative Medicine after Spinal Cord Injury: In Vitro and In Vivo Study

**DOI:** 10.3390/ijms21197031

**Published:** 2020-09-24

**Authors:** Zhuo-Hao Liu, Yin-Cheng Huang, Chang-Yi Kuo, Chao-Ying Kuo, Chieh-Yu Chin, Ping K. Yip, Jyh-Ping Chen

**Affiliations:** 1 Department of Neurosurgery, Chang Gung Memorial Hospital, Linkou, Chang Gung University School of Medicine, Kwei-San, Taoyuan 33305, Taiwan; b8402022@gmail.com (Z.-H.L.); ns3068@gmail.com (Y.-C.H.); recall04729@gmail.com (C.-Y.K.); gobananas929@gmail.com (C.-Y.C.); 2 Department of Chemical and Materials and Materials Engineering, Chang Gung University, Kwei-San, Taoyuan 33302, Taiwan; onesky1997@gmail.com; 3 Queen Mary University of London, Barts and The London School of Medicine and Dentistry, Blizard Institute, Centre for Neuroscience, Surgery & Trauma, London E1 2AT, UK; p.yip@qmul.ac.uk; 4 Department of Plastic and Reconstructive Surgery and Craniofacial Research Center, Chang Gung Memorial Hospital, Linkou, Kwei-San, Taoyuan 33305, Taiwan; 5 Research Center for Food and Cosmetic Safety, Research Center for Chinese Herbal Medicine, College of Human Ecology, Chang Gung University of Science and Technology, Taoyuan 33302, Taiwan; 6 Department of Materials Engineering, Ming Chi University of Technology, Tai-Shan, New Taipei City 24301, Taiwan

**Keywords:** nanofiber, spinal cord injury, docosahexaenoic acid, regenerative medicine, poly(lactic acid)

## Abstract

Spinal cord injury (SCI) is associated with disability and a drastic decrease in quality of life for affected individuals. Previous studies support the idea that docosahexaenoic acid (DHA)-based pharmacological approach is a promising therapeutic strategy for the management of acute SCI. We postulated that a nanostructured material for controlled delivery of DHA at the lesion site may be well suited for this purpose. Toward this end, we prepare drug-loaded fibrous mats made of core-shell nanofibers by electrospinning, which contained a polylactic acid (PLA) shell for encapsulation of DHA within the core, for delivery of DHA in situ. In vitro study confirmed sustained DHA release from PLA/DHA core-shell nanofiber membrane (CSNM) for up to 36 days, which could significantly increase neurite outgrowth from primary cortical neurons in 3 days. This is supported by the upregulation of brain-derived neurotropic factor (BDNF) and neurotrophin-3 (NT-3) neural marker genes from qRT-PCR analysis. Most importantly, the sustained release of DHA could significantly increase the neurite outgrowth length from cortical neuron cells in 7 days when co-cultured with PLA/DHA CSNM, compared with cells cultured with 3 μM DHA. From in vivo study with a SCI model created in rats, implantation of PLA/DHA CSNM could significantly improve neurological functions revealed by behavior assessment in comparison with the control (no treatment) and the PLA CSNM groups. According to histological analysis, PLA/DHA CSNM also effectively reduced neuron loss and increased serotonergic nerve sprouting. Taken together, the PLA/DHA CSNM may provide a nanostructured drug delivery system for DHA and contribute to neuroprotection and promoting neuroplasticity change following SCI.

## 1. Introduction

Being a very debilitating pathology, spinal cord injury (SCI) may lead to irreversible sensory, motor, and autonomic disabilities in most cases. To date, SCI treatment remains one of the most challenging problems in regenerative medicine with no effective cure. The pathological and pathophysiological changes after SCI include primary injury and subsequent secondary injury [1,2]. As the pathophysiological mechanism of SCI is more than simple mechanical disruption of nerve transmission, multiple treatment interventions have been suggested to fully improve neurological function following SCI. Previous studies have suggested that therapeutic strategies such as bridging the lesion, drug delivery, and cell delivery could be effective for the treatment of SCI. Nonetheless, although direct administration of pharmacological agents or neurotrophic factors to the injury site in SCI is frequently applied, such approaches did not lead to favorable outcomes due to the rapid biological clearance of these agents from the body [3,4,5]. The nanotechnology-based drug delivery system to the central nervous system (CNS) may provide new opportunities for SCI repair [6].

After adipose tissue, the CNS has the second-highest concentration of lipids, with a high concentration of lipids in the brain being comprised of polyunsaturated fatty acids (PUFAs) [7,8]. Omega-3 PUFAs are important components of cell membranes and are precursors to many other substances in the body, such as those involved in regulating blood pressure and inflammatory responses. The major omega-3 PUFAs in the CNS is docosahexaenoic acid (DHA), composing 10–20% of such fatty acids [9]. Normal CNS function and structure have been proposed to be dependent on an optimal level of omega-3 PUFAs, and if reduced, may lead to neurologic deficits [10] or cognitive changes [11]. Using the transient spinal cord ischemia model in rats, Lang-Lazdunski et al. were the first group to show the beneficial effect of omega-3 PUFAs after SCI [12]. Over the past 10 years, there has been increasing interest in the health benefits of PUFAs, with emerging evidence support that omega-3 PUFAs have significant therapeutic potential in a variety of CNS disorders, including Zellweger syndrome, schizophrenia, depression, and Alzheimer’s disease [13]. Most importantly, a number of clinical and pre-clinical studies have shown the neuroprotective effects from diets rich in omega-3 PUFAs [14,15,16].

The route of administration of DHA after SCI is important for its potential clinical applications. Previous studies have reported that after intravenous injection of radiolabelled fatty acids, PUFAs showed a fast clearance rate from plasma with a half-life less than 1 min. In addition, only 1% of the injected dose was incorporated into brain lipids, particularly phospholipids [17,18,19]. Accordingly, efficient administration strategies for protecting these highly unsaturated fatty acids against oxidation in addition to their sustained delivery through controlled drug release are necessary in order to avoid the loss of therapeutic effects. In this sense, the electrospinning process is a straightforward and versatile drug encapsulation technique suitable for the production of nanofibers/microfibers containing bioactive compounds. Indeed, lipophilic compounds such as β-carotene and fish oil have been successfully entrapped into nanofibers produced by electrospinning of a zein polymer solution prepared in water/ethanol solutions [20,21].

The coaxial electrospinning is an attractive process that contains a concentric spinneret accommodating two different solutions simultaneously for producing core-shell nanofiber structure with high quality and improved functionality [22]. During coaxial electrospinning, a core solution that usually contains bioactive substances such as drugs, antibiotics, or proteins in solution form, is delivered through the inner needle. The shell solution, usually containing polymeric materials dissolved in an organic solvent, is delivered through the outer needle. Under the action of an applied electric field to the tip of the needle, a liquid jet is ejected from the needle tip above a threshold voltage. After solvent evaporation, core-shell structure nanofibers can be collected with a conductive collector placed at a suitable distance from the needle tip [23]. Core-shell nanofibers have been suggested as controlled drug delivery carriers with several advantages over monolithic nanofibers, such as drug/protein incorporation efficiency in the nanofibers, control of drug release rate, as well as maintenance of structure and activity of bioactive substance [24]. As high and frequent dosing is required to enhance the performance of drugs for SCI repair due to the low intrinsic potency and short half-lives when manipulated in vivo, core-shell nanofibers look attractive as a drug delivery system to achieve a rapid functional recovery of the spinal cord after SCI [25].

Using biodegradable polymers for sustained drug delivery and other biomedical applications has attracted growing attention recently [26]. Consider polymers to be used to prepare DHA-loaded core-shell nanofibers to bridge SCI, polylactic acid (PLA) could be a promising candidate due to its outstanding properties like nontoxicity, biodegradability, and biocompatibility. Indeed, PLA has been considered as one of the most promising biodegradable materials to be widely studied and reported for medical use [27]. The slow degrading nature of PLA is also important for SCI repair in vivo as long periods (3–6 months) are usually required for functional regeneration after SCI.

**Figure 1:**
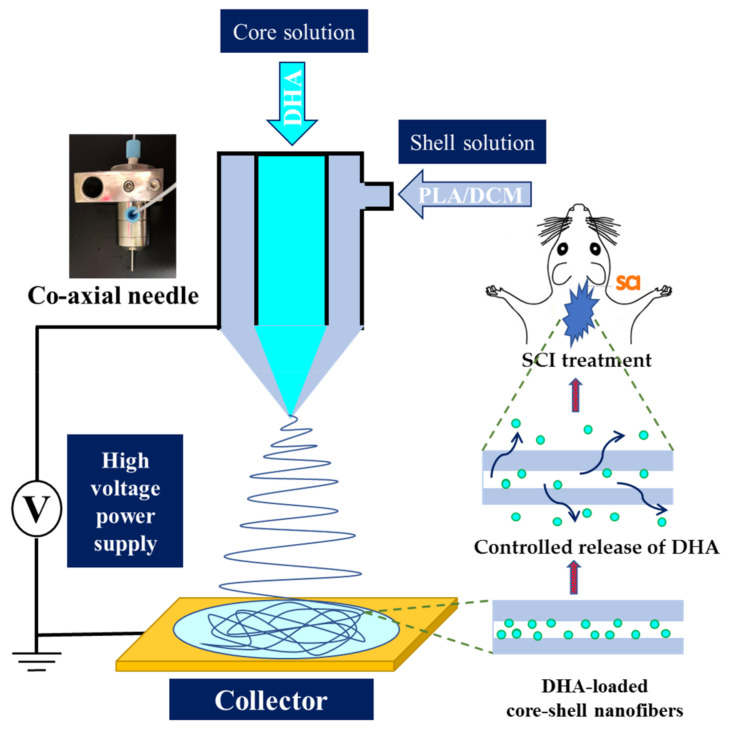
The schematic diagram showing the preparation of docosahexaenoic acid (DHA)-loaded core-shell nanofibers using polylactic acid/dichloromethane (PLA/DCM) shell solution for the treatment of spinal cord injury (SCI) in rats.

In this study, we intend to use coaxial electrospinning to prepare a DHA-loaded core-shell nanofiber membrane (CSNM) for restoring functional loss after SCI, using PLA as the base polymer (Figure 1). The effects of electrospinning parameters on nanofiber morphology were studied. The optimum PLA/DHA CSNM was characterized for mechanical properties and drug release profile. Finally, efficacies of prompting neurite outgrowth in vitro, and recovery of neurologic function with PLA/DHA CSNM treatment in vivo, using the SCI model in a rat model, were studied.

## 2. Results

### 2.1. Preparation and Characterization of Core-Shell Nanofiber Membrane (CSNM)

**Figure 2:**
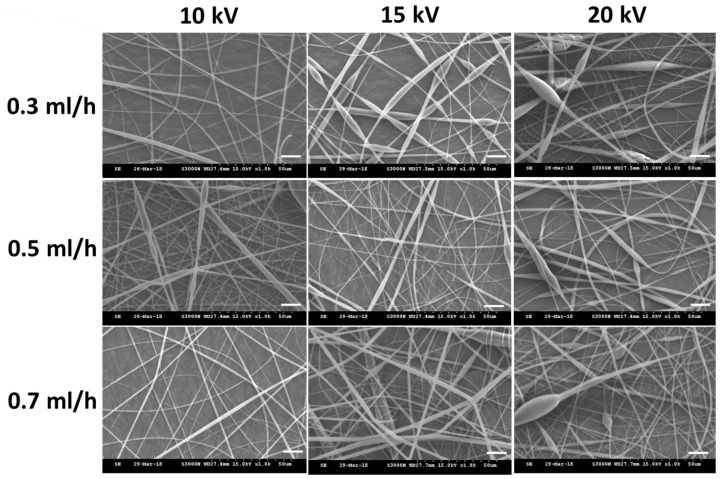
The scanning electron microscope (SEM) images of the electrospun polylactic acid/docosahexaenoic acid (PLA/DHA) core-shell nanofibers fabricated under different voltage (in kV) and core solution flow rate (in mL/h).

We used a 10% (*w/v*) PLA shell solution prepared in dichloromethane (DCM) and a 1 mM DHA core solution (prepared in ethanol) for the fabrication of PLA/DHA CSNM. The voltage and flow rate during electrospinning were reported to be major parameters affecting the morphology and diameter of electrospun nanofibers [28]. Therefore, we studied the effect of voltage and core solution flow rate on nanofiber morphology with a constant shell solution flow rate (1 mL/h). The range of chosen parameters for electrospinning was determined beforehand from pilot runs for the successful formation of the nanofiber membrane. As shown from images taken with a scanning electron microscope (SEM) in Figure 2, all runs generate nanofibers free of beads-on-string morphology. Although stable nanofiber formation was confirmed within the range of chosen voltage and flow rate, the nanofibers tended to become more irregular and with more uneven morphology at higher voltages (15 kV and 20 kV). Considering the rate of fabricating CSNM, we chose 10 kV and the highest flow rate (0.7 mL/h) to fabricate PLA/DHA CSNM for the following studies, which could provide bead-free nanofibers under the well-controlled electrospinning condition. The average fiber diameter was calculated to be 644 ± 106 nm from SEM images. The membrane thickness was 136 ± 10 μm and the loading of DHA was 6.23 ± 0.43 μg/mg CSNM after dissolving the membrane in dichloromethane (DCM) and quantified DHA concentration with an enzyme-linked immunosorbent assay (ELISA) kit.

**Figure 3:**
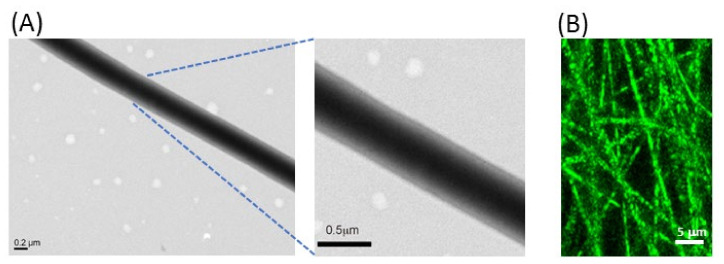
The transmission electron microscope (TEM) (**A**) and the confocal laser scanning microscope (the core solution was labeled with fluorescein isothiocyanate) (**B**) micrographs of polylactic acid/docosahexaenoic acid (PLA/DHA) core-shell nanofibers.

As demonstrated in Figure 3A, the formation of distinguishable core-shell nanofiber structure could be elucidated by observation under a transmission electron microscope (TEM). Indeed, a sharp boundary separating the shell from the core was confirmed from the TEM image of a single PLA/DHA nanofiber, with ~300 nm core diameter and ~80 nm shell thickness. To confirm the DHA encapsulation within the CSNM, a fluorescence dye (fluorescein isothiocyanate) was added to label the core solution, followed by the same electrospinning condition to prepare fluorescently labeled nanofibers. As shown in Figure 3B, nanofibers examined by confocal laser scanning microscopy revealed a fluorescent signal within the nanofibers, supporting the successful formation of core-shell nanofibers.

**Figure 4:**
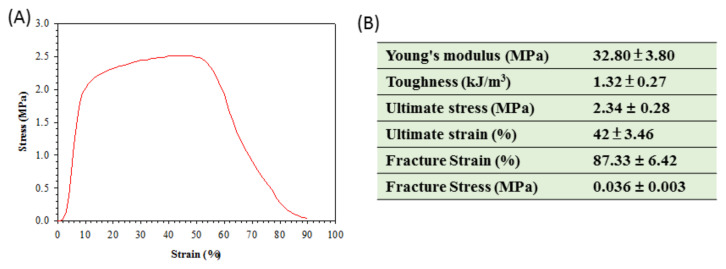
A typical tensile stress–strain curve of polylactic acid/docosahexaenoic acid (PLA/DHA) core-shell nanofiber membrane (**A**) and parameters of mechanical property determined from the stress-strain curves (*n* = 6, mean ± SD) (**B**).

The mechanical properties of a membrane intended for the treatment of SCI is an important design criterion, as matching the mechanical properties of a material to the surrounding native tissue may facilitate cell migration [5]. Therefore, the mechanical properties of PLA/DHA CSNF were fully characterized by tensile mechanical testing. As shown from Figure 4A, a stress-strain curve typical for a ductile material was found for PLA/DHA CSNF, from which parameters of the mechanical property was calculated (Figure 4B). The Young’s modulus is a mechanical property that measures the stiffness of a material and calculated form the slope in the stress-strain curve. The toughness represents a material’s resistance to fracture when stressed and is determined from the area under the stress-strain curve.

**Figure 5:**
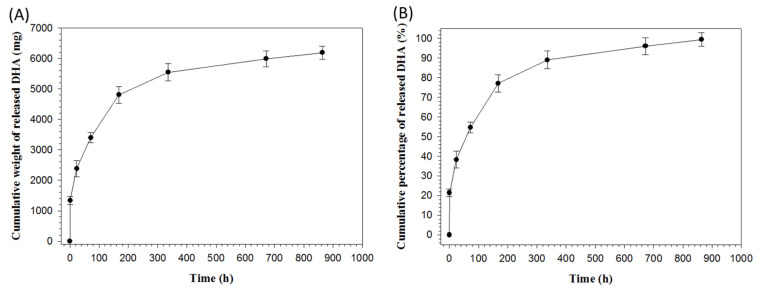
The release profiles of docosahexaenoic acid (DHA) from polylactic acid/docosahexaenoic acid (PLA/DHA) core-shell nanofiber membrane determined from the cumulative released weight (**A**) and cumulative released percentage (normalized to loaded DHA in the membrane) (**B**) of DHA.

The release profile of PLA/DHA CSNM shows the initial burst release of DHA from the membrane, followed by sustained drug behavior extending up to 36 days (Figure 5A). The cumulative DHA release percentage was 55% within 72 h and reached quantitative release at the end of the drug release experiment (Figure 5B).

### 2.2. In Vitro Cell Culture

**Figure 6:**
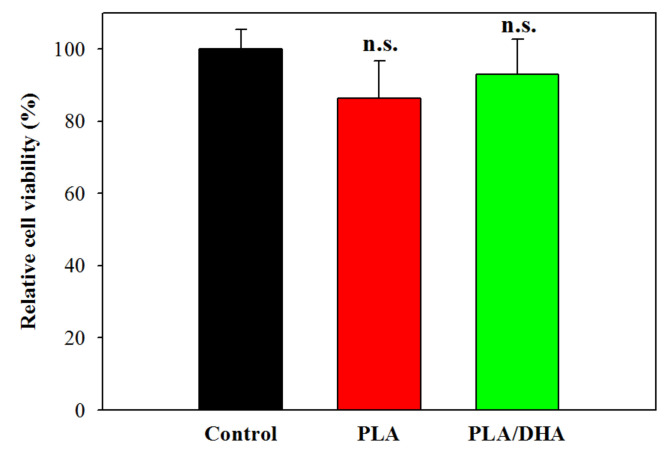
Evaluation of the cytotoxicity of core-shell nanofiber membrane toward fibroblasts using the 24 h extraction medium of polylactic acid (PLA) or polylactic acid/docosahexaenoic acid (PLA/DHA) core-shell nanofiber membrane for the culture of fibroblasts (*n* = 6, mean ± SD). The control is cells cultured with fresh cell culture medium. The cell viability was determined using the MTS assay and normalized to the control. n.s., not significant compared to control (*p* >0.05).

Evaluation of the possible cytotoxicity of CSNMs was carried out according to the ISO10993-5 standard for a medical device using the 24 h extract of a CSNM for cell culture. A PLA CSNM was prepared using the same electrospinning parameters but without DHA in the core solution for comparison. Using 3T3 fibroblasts, the relative cell viability was determined for PLA and PLA/DHA CSNM and compared with cells cultured in fresh cell culture medium (control). The rationale behind the experiment was to analyse whether any of the constituents of the CSNMs or residual organic solvent may have cytotoxic effects. As shown in Figure 6, the relative cell viability of PLA or PLA/DHA is not significantly different from that of the control, endorsing the high biocompatibility of the CSNM fabricated by electrospinning.

**Figure 7:**
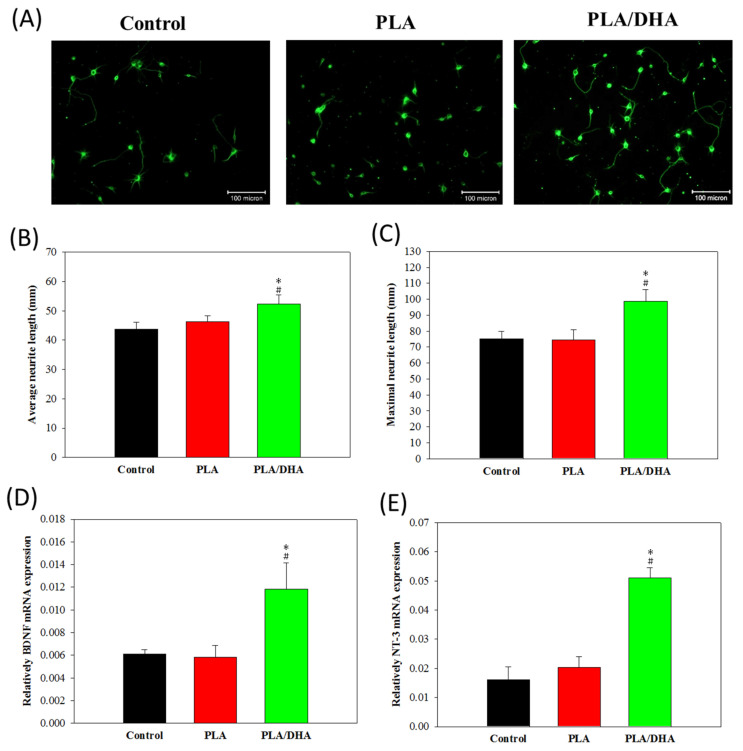
The neurite outgrowth was observed from β-tubulin III-positive primary cortical neuron cells under a fluorescent microscope after being co-cultured with polylactic acid (PLA) or polylactic acid/docosahexaenoic acid (PLA/DHA) core-shell nanofiber membrane in cell culture medium for 3 days (bar = 100 μm) (**A**). The control was in the culture medium only. The average neurite length (**B**) and the maximum neurite length from neurite outgrowth was calculated for each neuron (**C**) using the ImageJ software (*n* = 3, mean ± SD). The relative mRNA expression of brain-derived neurotropic factor (BDNF), (**D**) and neurotrophin-3 (NT-3) (**E**) neural marker gene was determined by real-time polymerase chain reaction (qRT-PCR). * *p* <0.05 compared with control, ^#^ *p* <0.05 compared with PLA.

To determinate whether DHA-load CSNMs directly affected the elongation of existing neurites, we characterized neurite outgrowth from primary cortical neuronal cells under different culture conditions. Neurons were stained with an antibody against β-tubulin-III, a specific marker for the somatodendritic compartment, 3 days after co-cultured with PLA or PLA/DHA CSNM in cell culture medium and compared with control (only cell culture medium) (Figure 7A). In the primary cortical neuron culture, the PLA/DHA group significantly increased the average neurite length per cell body (Figure 7B) and showed greater maximum neurite length per neuron (Figure 7C) when compared with PLA and control groups. No significant difference was noted between the PLA and control groups. This suggests that PLA/DHA CSNM can increase axonal growth and modulate neurogenesis. In order to explore the underlying mechanism that PLA/DHA CSNM promotes neurite outgrowth, primary cortical neurons co-cultured with the membrane for 3 days were subjected to quantitative real-time polymerase chain reaction (qRT-PCR) analysis of gene expression. As shown in Figure 7D,E, the relative mRNA expression of neural marker gene brain-derived neurotropic factor (BDNF) and neurotrophin-3 (NT-3) were significantly higher in PLA/DHA group compared with the other groups, with no difference found between PLA and control groups. This endorsed the activity of released DHA from PLA/DHA CSNM, which could enhance the neurite outgrowth demonstrated in Figure 6A–C, through modulation of BDNF and NT3 gene expression.

**Figure 8:**
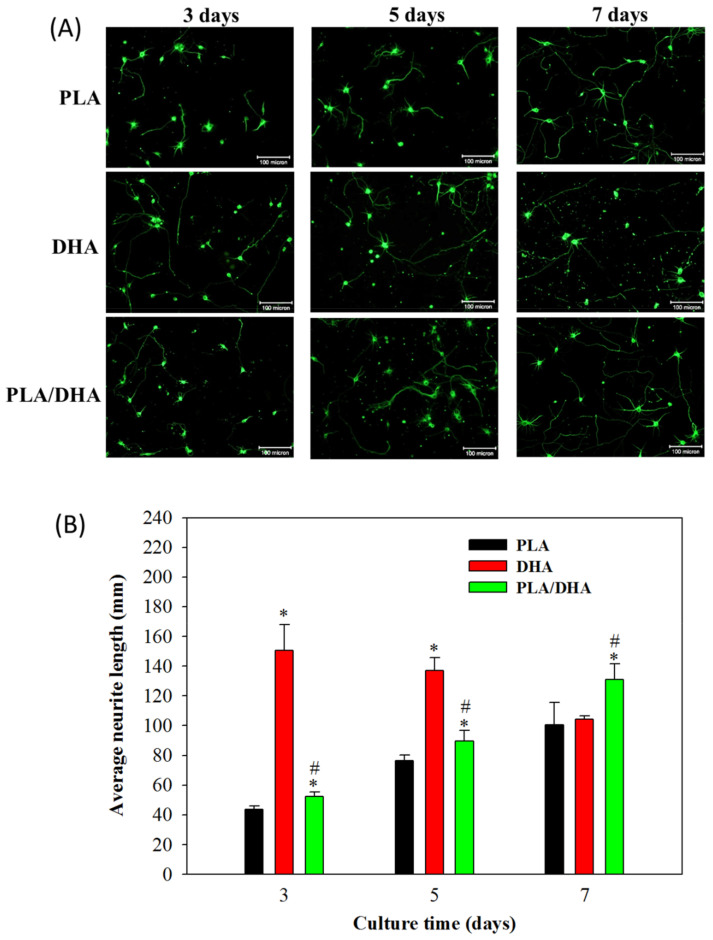
(**A**) The observation of neurite outgrowth from β-tubulin-III-positive primary cortical neuron cells after being co-cultured with polylactic acid (PLA) and polylactic acid/docosahexaenoic acid (PLA/DHA) core-shell nanofiber membrane in culture medium or cultured with 3 μM DHA in culture medium for 3, 5, and 7 days (bar = 100 μm). (**B**) The average neurite length due to neurite outgrowth was calculated for each neuron (*n* = 3, mean ± SD). * *p* <0.05 compared with control, ^#^ *p* <0.05 compared with PLA.

To evaluate the advantage of using PLA/DHA CSNM for temporally and spatially controlled DHA delivery, we further evaluated the time-lapsed neurite outgrowth pattern from primary cortical neuron cells after being co-cultured with PLA/DHA or PLA CSNM in cell culture medium, as well as in cell culture medium supplement with 3 μM DHA. Primary cortical neuron cells co-cultured with PLA/DHA CSNM presented fewer aggregates than cultured in the presence of free DHA (Figure 8A). However, continuous neurite outgrowth was identified in cortical neuronal cells co-cultured with PLA/DHA CSNM. Significant neurite outgrowth was observed on day 7 among all three groups. Regardless of elapsed culture time, cortical neuron cells co-cultured with PLA/DHA CSNM showed significantly increased average neurite length compared with PLA CSNM. The difference in average neurite length between PLA and PLA/DHA also appeared to increase with culture time, supporting DHA released from PLA/DHA CSNM remains active to promote neurite outgrowth with time (Figure 8B). Comparing PLA/DHA and DHA groups, although the average neurite length was lower for PLA/DHA up to day 5; the trend was reversed on day 7, when the neurite length of PLA/DHA was significantly higher than DHA. This difference underlined the advantage of using PLA/DHA CSNM for controlled DHA delivery over a 3 μM DHA treatment in vitro for neurite outgrowth. Taken together, our results support that the spatio-temporal DHA delivery with PLA/DHA CSNM can increase axonal growth and modulate neurogenesis in vitro.

### 2.3. In Vivo Study

**Figure 9:**
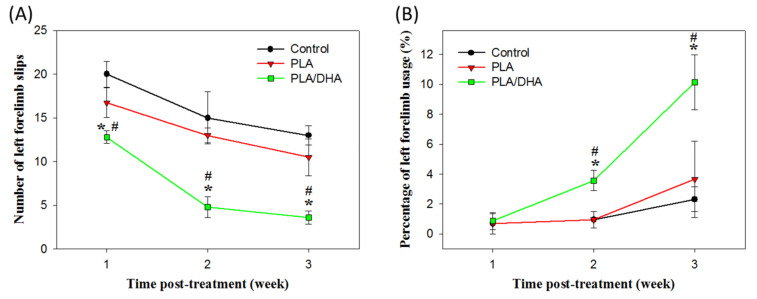
The evaluation of the neurological function recovery by the grid exploration test (**A**) and the cylinder test (**B**) using a spinal cord injury model in rats. The lesion was covered with a polylactic acid (PLA) or a polylactic acid/docosahexaenoic acid (PLA/DHA) core-shell nanofiber membrane for treatment. The control group received no treatment. * *p* <0.05 compared with control, ^#^ *p* <0.05 compared with PLA.

The grid exploration test has been reported to be sensitive enough to evaluate the sensorimotor coordination of the four limbs in the SCI animal model [29,30]. It was used here for the behavioral assessment of rats in control, PLA CSNM-treated, and PLA/DHA CSNM-treated groups, for their ability to accurately place the denervated forepaws following the spinal cord lesion. As shown in Figure 9A, the number of left forelimb slips of the tested animal was significantly less in the PLA/DHA group than the PLA and control groups as early as 1-week post-treatment and showed the same trend thereafter until the end of the experiment. By 3 weeks post-treatment, the number of foot-slips being made was 3.6 ± 0.7 in the PLA/DHA group compared to 10.5 ± 1.2 in the PLA group and 13.0 ± 0.7 in the control group (Figure 8A). From these data, PLA/DHA CSNM shows a significant effect on the neurological recovery of animals with SCI from the analysis of left forelimb slips.

Another behavioral assessment was from the cylinder test that measured a rodent’s spontaneous forelimb use, which can then be used to evaluate the sensory-motor function in a number of injury models that cause forelimb use to be asymmetrical [31]. At one week following spinal lesion surgery, a marked decrease in the forelimb usage for weight support during vertical exploration of a cylinder was noted for all groups (Figure 9B). However, at 2 and 3 weeks after surgery, the percentage of forelimb usage with the left (lesioned side) forelimb in PLA/DHA group significantly increases over those in PLA and control groups, with no significant difference found between these groups. This pattern indicated that the animals receiving PLA/DHA CSNM implantation could bear weight on the left forelimbs more frequently; supporting that PLA/DHA CSNM treatment improves neurological function recovery after SCI.

**Figure 10:**
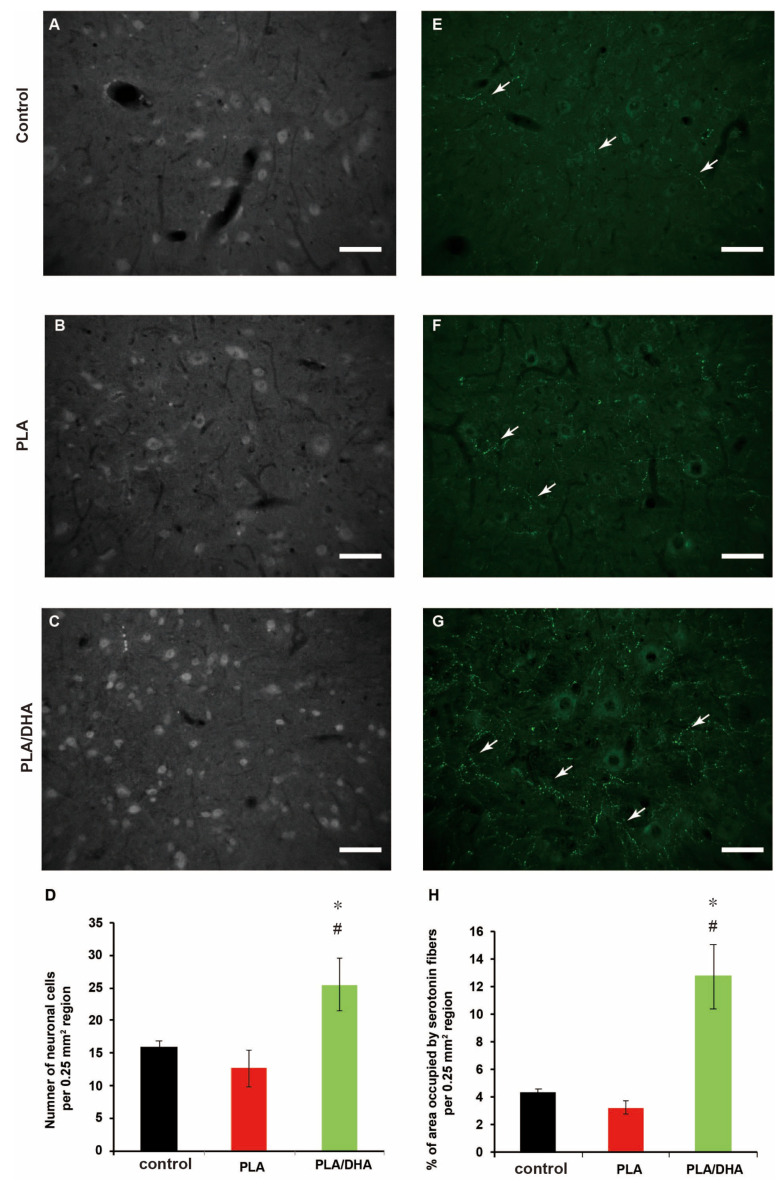
The polylactic acid/docosahexaenoic acid (PLA/DHA) core-shell nanofiber membrane (CSNM) prevented neuronal nuclei (NeuN) immunopositive cell loss and increase serotonin (5-HT) positive neurofiber sprouting in the rat after spinal cord injury. (**A–C**) The sections caudal to the injury site of the contused rat spinal cord were immunostained for NeuN and analyzed (bar = 100 μm). (**D**) The PLA/DHA CSNM implanted rats show significantly increased NeuN immunolabelled cells when compared to control and PLA groups at 21 days post-injury. (**E–G**) The sections show serotonergic fibers (white arrows) in the spinal cord tissue caudal to the lesion site after staining with anti-serotonin antibody (bar = 100 μm). (**H**) The quantification of serotonergic fibers reveals the serotonergic fiber is significantly increased in rats receiving PLA/DHA CSNM implantation. * *p* <0.05 compared with control, ^#^ *p* <0.05 compared with PLA.

From histological analysis, the application of PLA/DHA CSNM leads to significantly improved neuronal survival over PLA CSNM 3 weeks post-spinal injury in rats, as demonstrated by the number of neurons immunostained for the neuronal marker neuronal nuclei (NeuN), around the injury epicentre (Figure 10A–C). In particular, a significantly increased number of neuron cells in PLA/DHA-treated rats compared to PLA-treated and control rats was observed (Figure 10D), which supports that PLA/DHA CSNM can provide protection against neuronal cell loss after a spinal cord lesion. To investigate neuroplasticity changes after treatment, the serotonergic fibers were identified by utilizing serotonin (5-HT) immunohistochemical staining (Figure 10E–G). The number of serotonin fibers was quantified as 5-mm caudal to the lesion site (white arrows). The quantification results reveal rubout sprouting and significantly more serotonergic fibers caudal to the lesion in animals received PLA/DHA CSNM treatment compared to PLA and control groups (Figure 10H).

## 3. Discussion

Various strategies have been explored to create adequate biomaterial for nerve regeneration. Electrospun nanofiber membrane may be useful in guiding neuron regeneration in the spinal cord and brain, as well as serving as a scaffold to differentiate and guide transplanted neurons and stem cells in nervous system lesions [32]. Toward the use of nanofiber membrane in nerve regeneration, the mechanical properties of PLGA/DHA CSNM could meet the end of a biomaterial for SCI repair, from both softness and mechanical strength (Figure 4) [33]. Many reports have shown the benefits of using DHA for inducing noticeable functional recovery after SCI [15,16,34,35]. The focus of such studies with omega-3 fatty acids after the CNS traumatic injury was mainly based on their neuroprotective potentials. Nonetheless, the benefits offered by DHA after a central neurological injury may also be related to its role in promoting neuroplasticity. Indeed, DHA has been reported to increase the maximum neurite length and the number of neurites in cortical neuron and embryonic hippocampal cultures [36,37], as well as in primary sensory neurons [38]. In animal studies, oral DHA has been shown to promote the synthesis of components of synaptic membranes and specific presynaptic and postsynaptic proteins [39] and to improve brain learning involving synaptic plasticity [40].

In order to achieve the controlled release of DHA in the lesion site, we develop core-shell structure nanofibers in this study by accommodating DHA within the core region. This design may modulate DHA release to address the needs in suppressing inflammatory responses at the early stage and promoting axon growth at the later stage after SCI. We found that most DHA was released during the first 3 days, followed by continuous and sustained DHA release up to 36 days (Figure 5). With a burst release of close to half of its encapsulated DHA, PLA/DHA CSNM may play a major role in attenuating early inflammation. Following the initial burst DHA release, a low dose of DHA delivery from the PLA/DHA CSNM may effectively stimulate axon growth.

The PLA/DHA CSNM was shown to favor neurite outgrowth from primary neurons on day 3 (Figure 7A–C). Consider the possible mechanisms underlying the effects of DHA on neuroplasticity. Although the exact mechanisms remain unknown, the reasons behind the effects of DHA on neurite outgrowth are likely to involve complex interactions of the structure, function, and gene expression of the neuronal membrane. As a general strategy to promote axonal sprouting and remodeling, the application of exogenous neurotrophic factors, such as BDNF and NT3, was used in several studies [41,42]. As BDNF and NT3 are constantly expressed in the cortex and spinal cord, respectively, they were reported to enhance axonal sprouting from the intact side after SCI in animal models [42,43]. In line with the molecular mechanism for axonal sprouting, only primary cortical neurons co-cultured with DHA/PLA CSNMs showed significantly up-regulated BDNF and NT3 gene expression (Figure 7D,E). Furthermore, not only did the PLA/DHA CSNM favor primary neuron cell growth, time-lapsed study indicated continued neurite outgrowth in neuron cells co-cultured with PLA/DHA CSNM, which even exceed the efficacy offered by a single dose of DHA treatment on day 7 (Figure 8). This endorses the importance of sustained DHA delivery to promote neurite outgrowth. Interestingly, a single dose of DHA treatment was found not to offer better neurite outgrowth compared to the control group at this time point (Figure 8).

In studies on rodent brain, DHA supplementation increased CaM-dependent protein kinase II (CaMKII) and cAMP-responsive element-binding protein (CREB) levels and BDNF secretion to strengthen synaptic plasticity for spatial learning memory formation [44,45]. The BDNF system seems crucial for mediating the action of DHA in the brain, as a diet deficient in DHA has been shown to reduce the activation of tropomyosin receptor kinase B (TrkB) receptors [46]. Furthermore, DHA may also activate transcription factors, such as peroxisome proliferator-activated receptors (PPARs) and retinoid X receptors (RXRs) by altering their gene expression. Previous studies have shown that DHA reduced monocyte chemoattractant protein-1 (MCP-1) expression and lipopolysaccharide-induced nuclear factor-kappaB (NF-κB) activation, through the activation of PPARs [47,48]. Activation of PPARs can also increase BDNF and nerve growth factor (NGF) levels [49] and induce neuronal differentiation by modulating the BDNF/TrkB pathway [50]. An increased BDNF level was also detected in traumatic brain injury (TBI) [51,52] and cerebral ischemia [53] animal models after DHA supplementation, which could be correlated to functional recovery in the neurological system. Overall, our data is in line with those findings, as shown from the up-regulated BDNF expression and neurogenesis from DHA.

From in vivo study, DHA/PLA treatment produced a marked recovery of neurological function, with significant improvement from 1 week (grid exploration test) and 2 weeks (cylinder test) onwards (Figure 10). In grid exploration tasks, the PLA/DHA group made few foot slips, but PLA-treated lesioned rats and control were severely and significantly impaired throughout testing, albeit with some recovery over time. In the cylinder test, the deficits in ipsilateral forelimb usage were about 1% among the three groups. However, 2 and 3 weeks following injury, the percentage of impair forelimb usage significantly increased compared with PLA and control groups. These findings are consistent with previous studies, which demonstrated DHA could enhance neurological recovery after SCI [30].

DHA was reported to reduce neuronal and oligodendrocyte loss, lesion size, and apoptotic death, which led to the improvement of neurological outcome from the motor score, general balance, and limb coordination [35,54]. In our study, the neuroprotective effect of PLA/DHA is demonstrated by the number of neurons immunostained for the neuronal marker NeuN, around the injury epicenter (Figure 10D). These results show that DHA released from PLA/DHA CSNM can protect against neuronal loss after a spinal cord lesion. In regard to neuroplasticity changes following SCI, several studies have previously reported that rapidly diminished spinal levels of serotonin occur ipsilateral to a spinal lesion following thoracic SCI and cervical SCI [30,55], which is correlated with the severity and recovery of SCI. In our animal study, quantitative analysis revealed a significant increase in the number of serotonin-labeled axons in PLA/DHA group in comparison with PLA and control groups (Figure 10H). These data indicate that serotonin sprouting might contribute to promote functional recovery following PLA/DHA CSNM treatment.

## 4. Materials and Methods

### 4.1. Preparation of Core-Shell Nanofiber Membrane (CSNM)

The PLA/DHA CSNM was prepared from two distinct solutions destined respectively for the shell or core part of a nanofiber. The shell solution was prepared by dissolving 0.5 g PLA (poly(L-lactide-co-D,L-lactide), L-lactide:D,L-lactide = 70:30, Resomer^®^ LR 706 S; Evonik, Essen, Germany) in 5 mL of DCM to make a 10% (*w/v*)) PLA solution. The core solution was prepared by dissolving 0.654 mg DHA (D2534; Sigma-Aldrich, St. Louis, MO, USA) in 100 mL absolute ethanol to make a 1 mM DHA solution. The CSNM were prepared by electrospinning with a coaxial spinneret at different flow rates (shell flow rate = 1 mL/h, core flow rate = 0.3, 0.5, or 0.7 mL/h). When a high voltage (10, 15, or 20 kV) was applied to the polymer solution, the electrostatic force overcomes the surface tension of the polymer solution at the tip of the needle to form a Taylor cone, which is further elongated into a fluid jet by ejecting the polymer solution from the tip of the needle. Nanofibers formed from the charged fluid jet after solvent evaporation were collected using a collector covered with aluminum foil placed 10 cm from the needle tip. The PLA CSNM was prepared as a control by following the same preparation condition used for PLA/DHA CSNM but with DHA-free core solution. To prepare fluorescently labeled PLA/DHA CSNM, fluorescein isothiocyanate (FITC) fluorescence dye was added to the core solution before electrospinning.

### 4.2. Characterization of Nanofiber Membrane

The surface morphology of CSNMs was observed using a scanning electron microscope (SEM) (S3000N; Hitachi, Tokyo, Japan). At least 100 fibers randomly chosen from 10 SEM images were selected to measure the fiber diameter. The thickness of all membranes was measured with a dial thickness gauge (TECLOCK SM-1201, Nagano, Japan). A transmission electron microscope (TEM) (JEM-1230, JEOL, Tokyo, Japan) was used to determine the core-shell structure by operating at 75 kV [56]. The fluorescently labeled PLA/DHA CSNM was observed under a confocal laser scanning microscope (LSM 510 Meta, Zeiss, Oberkochen, Germany) at 364 nm/460 nm excitation/emission wavelength. The tensile properties of CSNM were evaluated using an H1KT mechanical testing machine (Tinius Olsen, Horsham, PA, USA) equipped with a 10 N loading cell. Samples were cut into strips (10 mm x 50 mm) and allowed to undergo tensile elongation at a crosshead speed of 0.3 mm/s and a gauge length of 30 mm. Young’s modulus (MPa), fracture strain (%), toughness (kJ/m^3^), ultimate stress (MPa) and ultimate strain (%) were calculated from the tensile stress-strain curves (*n* = 6). To determine the release profile of DHA from PLA/DHA CSNM, the membrane was cut into small pieces, and ~10 mg of sample was immersed in 1 mL of pH 7.4 phosphate buffer solution (PBS) at 37 °C. At predetermined time points, the PBS was completely removed for analysis and 1 mL of fresh PBS was added for continuous incubation up to 36 days. The amount of DHA released at each time point was determined by using an enzyme-linked immunosorbent assay (ELISA) kit for DHA (CEO623Ge, Cloud-Clone Co., Katy, TX, USA), which can detect 12.35–1000 pg/mL DHA based on a competitive inhibition test method. By following the manufacturer suggested procedure, we used 50 µl sample solution and determined the absorbance at 450 nm using an ELISA plate reader (Synergy HT, BioTek, Winooski, VT, USA). By recording the released weight of DHA at each time point, the cumulative released weight of DHA vs. time was reported. The cumulative released percentage of DHA was also calculated after normalizing to the amount of DHA loaded in 10 mg PLA/DHA CSNF. The amount of loaded DHA was determined from completely dissolved PLA/DHA CSNF in DMF for quantification of DHA concentration using a DHA ELISA kit as described before.

### 4.3. Primary Cortical Cell Culture

Primary cortical neurons were prepared from 18-day-old embryos of Sprague-Dawley rats following protocols approved by the Chang Gung University’s Institutional Animal Care and Use Committee [57]. A plastic dish (60 mm diameter) treated with polyethyleneimine was used for cell culture. The plating medium is Minimum Essential Medium (MEM) with Earle’s balanced salts and supplemented with 10% fetal bovine serum (FBS), 1 mM pyruvate, 1 mM L-glutamine, 26 mM sodium bicarbonate and 20 mM KCl and (pH 7.2). After 3 to 4 h for cell attachment, the plating medium was replaced with the neurobasal medium containing B-27 supplements, 2 mM L-glutamine, 1 mM 4-(2-hydroxyethyl)-1-piperazineethanesulfonic acid (HEPES) and 0.001% gentamycin sulfate for cell culture.

### 4.4. Cytotoxicity Evaluation

Cytotoxicity of the CSNMs was examined by the indirect contact method following the biological evaluation of medical devices in ISO 10993-5 [58]. Square shaped CSNMs (1 cm × 1 cm) were sterilized by UV light and immersed in 1 mL of Dulbecco’s Modified Eagle Medium (DMEM) containing 10% (*v/v*) FBS for 24 h. The extraction medium was removed and used for cell culture. 3T3 fibroblasts at 1 × 10^4^ cells per well were seeded in a 24-well tissue culture plate for 4 h and cultured with the extract medium for 24 h at 37 °C in a humidified 5% CO_2_ environment. Cells cultured with fresh cell culture medium were adopted as the control. The relative cell viability was determined from MTS assay using CellTiter 96^®^ AQueous One Solution Cell Proliferation Assay from Promega (Madison, WI, USA)). The solution absorbance at 492 nm was obtained from an ELISA plate reader (Synergy HT, BioTek, Winooski, VT, USA) and normalizing to that of control.

### 4.5. Neurite Outgrowth Evaluation

To evaluate the ability of PLA/DHA CSNM to promote neurite outgrow, primary cortical neuronal cells were re-suspended to a cell concentration of 2.5 × 10^5^ cells/mL after washing and pelleting, and seeded onto poly-D-lysine coated glass coverslips placed in a 24-well plate for culture in 5% CO_2_ incubator at 37 °C (*n* = 3). After culture for 1 day, a CSNM (1 cm × 1 cm) was placed at the bottom of a Corning Transwell^®^ cell culture plate insert (8.0 μm pore size porous membrane) for diffusion of DHA from the CSNM to the seeded cells on the well surface. The CSNM-loaded inserts were fitted into the 24-well culture plate, and the cell culture medium was added to fully immerse the membrane for co-culture of PLA/DHA CSNM with primary cortical neuron cells. Primary cortical neuronal cells cultured with cell culture medium, cell culture medium supplement with 3 μM DHA and co-cultured with PLA CSNM were used for comparison. After culture for 3, 5 or 7 days, the cells were fixed with 4% paraformaldehyde for 20 min, permeabilized with cold methanol and washed with PBS. For immunostaining, cortical neuronal culture was incubated at room temperature for 2 h with anti-β tubulin III primary antibody (Sigma-Aldrich, 1:1000). After staining with AlexaFluor 488 conjugated secondary antibody (Invitrogen, 1:1000) at room temperature for 45 min and PBS washing, cells were mounted with FluorSave reagent (Calbiochem) and observed under an inverted fluorescent microscope. Neurite length was analyzed by drawing a line along every neurite for each neuron cell body and analyzed using the ImageJ software (version 1.41, NIH, Bethesda, MD, USA) to calculate the average and maximum neurite length.

### 4.6. qRT-PCR to Detect BDNF and NT-3 mRNA Expression

To examine axonal outgrowth-related gene expression, the primary cortical neuron cells co-cultured with PLA and PLA/DHA CSNM for 3 days were subject to quantitative real-time polymerase chain reaction (qRT-PCR) analysis and compared with control runs using culture medium only. The expression of neural marker gene brain-derived neurotropic factor (BDNF) and neurotrophin-3 (NT-3) was analyzed using standard protocols for RNA isolation and cDNA synthesis. Total RNA was extracted from the primary cortical neuron cells using TRIzol^®^ reagent (Invitrogen) and reversely transcribed into cDNA by using Total RNA Isolation Kit and Maxime RT PreMix Kit according to the manufacturer’s protocols. The qRT-PCR was conducted using an SYBR Green RT-PCR kit in a MiniOpticon^TM^ real-time PCR detection system (Bio-Rad CFD-3120). Glyceraldehyde 3-phosphate dehydrogenase (GAPDH) acted as the housekeeping gene, and relative mRNA expression of BDNF and NT-3 was determined using the $2^{-\Delta \Delta \mathrm{ct}}$ relative quantification method. The primers used are listed in Table 1.

### 4.7. Animal Surgery

Cervical lateral hemisection of the spinal cord was performed in adult female Sprague-Dawley (SD) rats (250–300 g) using methods previously described [30,59]. All animal experiments were conducted according to protocols approved by the Chang Gung University’s Institutional Animal Care and Use Committee. Briefly, animals were anesthetized with isoflurane, and a dorsal midline incision was made at cervical level, to expose the cervical vertebrae. A left hemilaminectomy was carried out at C4 and C5, and then a lateral hemisection was made at the C4–5 level using a microblade. A CSNM was put over the lesion site and cover with dura matter. After surgery, the muscles and skin layers were sutured and animals were returned to a warm incubator for recovery. The control animal received only hemisection of cervical spinal without implantation of CSNM. Post-operative care involved sub-cutaneous injection of analgesic and saline twice daily for 3 days following surgery.

### 4.8. Grid Exploration Test

Animals were placed on a 20-cm raised grid platform (40 cm × 60 cm) containing 5 cm × 5 cm square grids every week post-operatively, to assess the misplaced steps made by the injured left forelimb. A misplacement was recorded when the limb protruded entirely through the grid and extended below the wire surface. An experimenter blinded to the treatment group scored the number of foot slips out of the first 30 steps taken with the left forelimb. All animals were tested for their baseline performance by subjecting them to the grid exploration test three times prior to surgery.

### 4.9. Cylinder Test

In this test, the animal was placed in a glass cylinder, and the number of times it rears up and touches the cylinder wall was measured. The footage was later analyzed in slow motion and the number of times the animal reached up and touched the wall with its left forelimb, right forelimb, or both forelimbs was counted. The results are expressed as the percentage of left forelimb usage for weight support relative to the total number of touches.

### 4.10. Histological Analysis

At the end of the study, animals were sacrificed and flushed with saline transcardially followed by 4% paraformaldehyde. After placing the spinal cord tissues in 20% sucrose for cryoprotection, spinal cord segments containing the hemisected region were cut horizontally at 20 μm using a cryostat and organized as slide series. Slides of the spinal cord section were randomly chosen and washed with gentle agitation in PBS. The sections were incubated in 10% normal donkey or goat serum for 30 min followed by an overnight incubation with primary antibodies (rabbit anti-serotonin, 1:3000; mouse anti-neuronal nuclei (anti-NeuN), 1:1000). The next day, sections were washed three times (5 min each) in PBS before being incubated for 2 h with secondary antibodies conjugated to Alexa Fluor 488 or 594 (1:1000). After another 5-min wash in PBS, sections were counterstained with the fluorescent nuclear dye Hoechst 33342 (2 mg/mL) for 5 min to facilitate the detection of cell nuclei. Slides were mounted in ProLong™ Gold antifade reagent.

### 4.11. Image Analysis of Histological Sections

Image analysis and quantification were carried out with the observer blinded to the group assignment. At least four sections per staining of the corresponding horizontal plane containing the region of interest (e.g., motor neurons) were viewed and captured on a Leica epifluorescence microscope at 20× magnification. The depth and distance from the lesion site of the images were taken and analyzed consistently for all animals. Quantitative analysis of the number of serotonin-labeled axons and the number of neuronal cells was conducted by randomly capturing images of the spinal cord 5-mm caudal to the lesion site. The immunoreactivity in captured images was then quantified in a 500 μm × 500 μm measuring frame by using the ImageJ analysis software. The level of immunoreactivity was expressed as the percentage of the area of the measuring frame that contained immunoreactivity.

### 4.12. Statistical Analysis

All data are expressed as mean ± standard deviation (SD). One-way analysis of variance (ANOVA) was used for statistical analysis using post-hoc Tukey HSD test. Significance was found when the *p*-value was less than 0.05.

## 5. Conclusions

We report successful fabrication of electrospun drug-loaded fibrous mats made of core-shell nanofibers, with PLA shell to encapsulate DHA within the core, for controlled release of DHA in situ. This PLA/DHA CSNM is endowed with adequate mechanical properties for sustained release of the biological cue DHA that has been proven to be effective for SCI repair. The PLA/DHA CSNM co-cultured with primary cortical neuron cells successfully promotes neurite outgrowth in vitro, which correlates well with the molecular mechanism of up-regulated neural marker genes BDNF and NT-3. Furthermore, the advantage of using CSNM for DHA delivery is also supported by increased neurite outgrowth length over a single dose of DHA treatment during primary cortical neuron culture. From the SCI model in rats, animals treated with PLA/DHA CSNM show pronounced neurological function recovery from behavior assessment within 1 or 2 weeks, which is supported by histological analysis. We thus believe this drug-loaded nanostructure may offer mechanical and chemical support for the fabrication of central nerve grafts in the future, either in vitro or in situ. This work may also provide a starting point for the further fabrication of other neural tissues by loading appropriate instructive cues.

## Figures and Tables

**Table 1:** Apoptotic primer sequence used for qRT-PCR analysis.

Genes	Forward Primer (5′→3′)	Reverse Primer (5′→3′)
NT-3^1^	CGTGGTGGCGAACAGAACAT	GGCCGATGACTTGTCGGTC
BDNF^2^	CTACGAGACCAAGTGCAATCC	AATCGCCAGCCAATTCTCTTT
GAPDH^3^	GCAAGTTCAAGGCACA	CATTTGATGTTAGCGGGAT

^1^ NT-3, neurotrophin-3; ^2^ BDNF, brain-derived neurotropic factor; ^3^ GAPDH, glyceraldehyde 3-phosphate dehydrogenase.
